# An Initial Cross-Cultural Comparison of Adult Playfulness in Mainland China and German-Speaking Countries

**DOI:** 10.3389/fpsyg.2018.00421

**Published:** 2018-03-29

**Authors:** Dandan Pang, René T. Proyer

**Affiliations:** ^1^Personality and Assessment, Department of Psychology, University of Zurich, Zurich, Switzerland; ^2^Personality and Assessment, Department of Psychology, Martin Luther University Halle-Wittenberg, Halle, Germany

**Keywords:** adult playfulness, cross-culture, situation-specific playfulness, positive traits, China

## Abstract

Compared with playfulness in infants and children, playfulness in adults is relatively under-studied. Although there is no empirical research comparing differences in adult playfulness across cultures, one might expect variations between Western and Eastern societies such as China. While playfulness is typically seen as a positive trait in Western culture, there are hints in Chinese culture that being playful has negative connotations (e.g., associations with laziness and seeing play as the opposite of work). The aim of this study was to compare expressions of playfulness in one sample from German-speaking countries (*n* = 143) and two samples from China (Guangzhou: *n* = 176; Beijing: *n* = 100). Participants completed one playfulness scale developed in the West (Short Measure of Adult Playfulness, SMAP) and one from the East (Adult Playfulness Questionnaire, APQ). Additional ratings of the participants were collected to measure: (a) the level of playful behavior expressed by people in different situations (e.g., when being around family members, in public, or on social media), and (b) individuals’ perceptions of society’s expectations concerning the appropriateness of being playful in the given situations. Overall, the results of the comparisons were mixed. Although SMAP scores did not vary significantly across the three samples, people from German-speaking countries tended to score higher on some facets of the APQ and some situational ratings. Stronger effects were found when comparing only the German-speaking sample and the Guangzhou sample. In addition to the cross-cultural differences that we expected, we also detected Chinese regional variations (North vs. South). We conclude that societal rules and cultural factors may impact expressions of playfulness in a society.

## Introduction

### Theoretical Background and Current Studies in Western Culture

Play, as a component of human behavior, is an innate part of our nature, and a basic *need to play* has been described as a core human characteristic that can take many forms, defined for instance as “to relax, amuse oneself, seek diversion and entertainment; to ‘have fun,’ to play games; to laugh, joke and be merry; to avoid serious tension” (Murray, 1938; p. 83). Developmental psychology has acknowledged the importance of play for the acquisition of different abilities and developmental transitions (Erikson, 1950; Piaget, 1951). Accordingly, infants and children have an intrinsic understanding of the importance of play (Yu et al., 2007). Previous studies suggest that play contributes to physical, cognitive, social, linguistic and emotional aspects of child development (Csikszentmihalyi, 1975; Lieberman, 1977; Isenberg and Quisenberry, 1988; Barnett, 1990; Blasi et al., 2002; Tamis-LeMonda et al., 2004; Ginsburg, 2007). For example, in cognitive development, play and games can assist children with creative thinking and behavioral flexibility (Piaget, 1951; Sutton-Smith, 1967), as well as widen their memory of factual knowledge (Lunzer, 1959). It has been argued that when we play, we are engaged in the purest expression of our humanity (Brown and Vaughan, 2009). Of course, play is not only limited to children. It can also be found in adults; even in comparatively serious situations (Bologh, 1976) such as when people are at work (Csikszentmihalyi, 1975; Csikszentmihalyi and LeFevre, 1989). For the present study, not only the actual behavior (*play*), but *playfulness* as a personality trait is of importance. Lieberman (1977) argues that “[…] playfulness as a quality of play would developmentally transform itself into a personality trait of the player in adolescence and adulthood” (Lieberman, 1977; p. 23).

Playfulness in adults is comparatively a rarely studied trait (Proyer, 2012a). It can be defined as: “[…] an individual differences variable that allows people to frame or reframe everyday situations in a way such that they experience them as entertaining, and/or intellectually stimulating, and/or personally interesting. Those on the high end of this dimension seek and establish situations in which they can interact playfully with others (e.g., playful teasing, shared play activities) and they are capable of using their playfulness even under difficult situations to resolve tension (e.g., in social interactions, or in work-type settings). Playfulness is also associated with a preference for complexity rather than simplicity and a preference for—and liking of—unusual activities, objects and topics, or individuals” (Proyer, 2017; p. 114). Previous research has shown that adult playfulness is associated with a large number of positive outcomes such as academic performance (Proyer, 2011); facilitation of positive emotions (Fredrickson, 2001); relationship satisfaction (Aune and Wong, 2002; Proyer, 2014b; Proyer et al., in press a); sexual selection (Chick, 2001; Chick et al., 2012; Proyer and Wagner, 2015); coping with stress (Qian and Yarnal, 2011; Magnuson and Barnett, 2013; Proyer, 2014a); and well-being (Barnett, 2012; Proyer, 2012c, 2013, 2014a)—to name but a few.

### Cross-Cultural Aspects and Current Studies in Eastern Culture

To the best of our knowledge, play and playfulness in adults are mostly studied from a Western perspective. For example, studies have recently been conducted with samples from the United States (Barnett, 2007), United Kingdom (Aroean, 2012), Denmark (Hasse, 2008), Switzerland (Proyer, 2011), and Germany (Proyer and Wagner, 2015), while Eastern countries have only rarely been studied (e.g., Yu et al., 2003, 2007; Yue et al., 2016). Studies conducted in German-speaking countries have led to the development of a multifaceted model of playfulness; i.e., other-directed, lighthearted, intellectual, and whimsical (Proyer, 2017). A discussion of structural issues (Proyer, 2012a; Proyer and Jehle, 2013) has provided support for its importance in academic settings (Proyer, 2011) and romantic relationships (Proyer, 2014a,b; Proyer et al., in press a), and its association with virtuousness and positive psychological functioning (Proyer and Ruch, 2011; Proyer, 2013). An analysis of German-speaking laypersons’ perceptions of how they use playfulness in their daily lives revealed seven main categories: well-being; humor and laughter; mastery orientation; creativity; relationships; coping strategies; and coping with specific situations (Proyer, 2014a). Overall, these findings provide support for the notion that people studied in German-speaking countries assign important functions to playfulness and that it is related to important outcome variables such as relationship satisfaction and academic success. Comparatively less knowledge exists about the role of playfulness in Eastern culture. In an effort to narrow this gap in the literature, the aim of this study was to compare measures developed in the West and the East, collect data from both Western culture (German-speaking countries) and Eastern culture (China), and see whether the findings differ.

This comparison is of particular interest since German-speaking countries are typically rated higher in *individualism* than China. On a 1 to 10-point scale, the country scores on Individualism-Collectivism are 7.90 for Switzerland, 7.35 for West Germany, 6.75 for Austria, and 2.00 for China (Suh et al., 1998). Hence German-speaking countries (Switzerland, Austria, and Germany) and China enable a cultural comparison along the Individualism-Collectivism dimension. People in individualistic countries display less conformity behavior (Hofstede, 2001; p. 236). One might argue that people in individualistic cultures utilize a larger variety of playfulness functions in different areas of life than those in collectivistic countries. For a better understanding of potential cultural differences, and given the absence of previous data, we discuss the Eastern perspective (Chinese, to be precise) on play and playfulness in more detail.

A common stereotype about the Chinese is that they are diligent (Smith, 1894). In one of the first chapters of his book “Chinese Characteristics” Smith (1894) concludes: “[…] there can be little doubt that casual travelers, and residents of the longest standings, will agree in a profound conviction of the diligence of Chinese” (p. 27). Smith also pointed out that this diligence is not characteristic of a single group within Chinese society, but rather that it can be applied to all residents of the country (Smith, 1894). Even nowadays, with a growing influence of globalization, it is still highly valued to be diligent in China. Aphorisms such as “
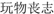
” (“Excessive attention to plaything saps the will”), “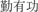, 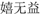
” (“Reward lies ahead of diligence, but nothing is gained by play”), and “
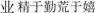
” (“Achievements are reached by hard work rather than play”), are taught to children when they start primary school. Overall, it seems as if many Chinese tend to have a negative bias toward play. One common belief is that play is the opposite of work [see Glynn and Webster (1992) for a Western representation of this idea] and is only reserved for children. Only by working hard, can happiness and success be achieved (Harrell, 1985). Of course, there are also other variables in addition to the individualism vs. collectivism dimension that may contribute to cultural differences; for example, the autonomy vs. embeddedness dimension in Schwartz’s (2006) theory of value orientation (for an overview see Sagiv et al., 2017). Playfulness shares characteristics with both intellectual and affective autonomy as it relates to intellectual striving and its pursuit enables positive experiences (e.g., Proyer, 2017). However, an emphasis on embeddedness does not seem to foster playful behaviors.

A dominant perception in Chinese culture seems to be that the intense competition of the education system requires their students to study hard without being distracted by play activities. There are only about twenty top-tier universities in mainland China and there are millions of students every year, all having only one chance in that year to get accepted via the national university entrance examination (Davey et al., 2007). It is also seen as one of the few chances for students from the rural areas in China to change their social class in a comparatively fast and low-cost way (Chen and Uttal, 1988). This competition forces children to study hard from the very first school day, so that they can get accepted into a better secondary school and eventually a better college. A big difference in comparison with competitive educational systems in the West (e.g., in the United States) seems to be that this national examination is the only criterion for Chinese high school students, whereas the United States system is characterized by a variety of criteria (i.e., in addition to achieving good grades, students are also encouraged to attend extracurricular activities). The idealized image of the hard-working student is culturally well-represented by paragons from earlier times. For example, a story tells us that Sun Jing (1425–1484), a student in Sichuan province, tied his hair to a house beam so that he could keep on learning and did not fall asleep despite his long working hours (Lin, 2012).

This relatively negative perspective on play and playfulness seems to have had an impact on the language, which has led to a basic problem for the present study. A term in Chinese that corresponds precisely to playfulness seems to be missing (see also Yu et al., 2007). It should be mentioned that the term “play” is avoided in the Chinese language in many cases. For example, instead of saying “playing football,” one says 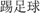 (kicking football),” while “playing the piano” is 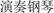 (performing on the piano).” Consequently, at the early stages of our study we asked Chinese students who study in Switzerland (and should have some understanding of the Western concept of play and playfulness) about their understanding and suggestions for translating the term “playfulness.” Twenty-two students (13 female, 9 male) were asked: “How would you translate the sentence ‘I am a playful person,’ especially the word ‘playful’?” The answers were diverse. Some of them referred to people as “not reliable,” “playboy,” “not nice,” or that it should be expressed as the “opposite of study” and so forth. It was mainly the students who had been abroad only for 1 year or less who expressed these associations. Those who had been abroad for more than 5 years had different opinions. They would link playful to adjectives like “humorous,” “witty,” or “interesting.” This may point to some cultural transmission in how playfulness is being perceived and in associations related to this individual differences variable (see also Barnett, 2017).

As mentioned above, China is a collectivistic country (Hofstede, 2001) with strong social hierarchies (Triandis et al., 1990; Markus and Kitayama, 1991). In Western culture, an individual’s dominant behavior is positively reinforced and people are encouraged to climb the hierarchy (Triandis and Gelfand, 1998). In contrast, a collectivistic society prefers subordination (Triandis and Gelfand, 1998) and praises agreeable individuals rather than dominant ones (Moskowitz et al., 1994; Realo et al., 1997). In this sense, play could be considered as not obeying certain rules and to being self-centered, which is not approved by collectivistic cultures and may even lead to anxiety and insecurity for those in power. As Confucius himself once remarked: “each should behave appropriately according to his or her station” and “man has to be serious to be respected” (cited after Liao, 2007).

It should be mentioned that there is an important differentiation between the public and the private self when discussing play and playfulness in Chinese culture and tradition. Confucius himself allows for *proper* playfulness, which refers to a form of private, moderate, good-natured, tasteful and didactically useful mirth (cited after Milner Davis and Chey, 2011). This sense of propriety can also be found in a famous quote by Pu Songling (1640–1715), a writer of the Qing dynasty, who notes: “There is no one who does not laugh, but one must laugh at an appropriate time” (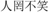, 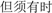; Liaozhai zhi yi, p. 155). Additionally, Daoism, as an alternative view of life, has a tradition of the appropriate use of playfulness. Two of the main pieces of Daoist literature, Liezi and Zhuangzi, are both made up of legends, jokes, parables and allegorical tales, all laced with playfulness and paradoxes. Daoists such as Zhuangzhou criticized Confucian social conventions by being a “huaji-ist,” “huaji” being an earlier indigenous term for humor. In addition, playfulness in China can also be found in many forms, both literary and conventional. For instance, Dayoushi (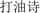), a Chinese literary game between friends where each player picks up a thought or expression from the last player and twists the meaning in an unexpected and, therefore, funny way, is one source of evidence (cited after Milner Davis and Chey, 2011). In the Chinese Spring Festival Gala, a wide variety of puns are found in the cross talk, since the Chinese language is rich in homophones. Western influences on humor seem to be comparatively limited. However, selected works (e.g., by Henri Bergson; see Milner Davis, 2014) were translated into Chinese and comparatively well-received in academic circles.

To summarize, although there are ambivalent attitudes toward playfulness, the negative perception of play and playfulness still seems to be present in China. Thus we expected that the Chinese participants in our study would be less playful than the German-speaking participants. Likewise, we expected that the differences in situations with hierarchical communication in various forms, such as in a public situation or at the workplace, would be larger. To assess the participants’ ratings of their level of playfulness in these different contexts, we developed a list of 14 different situations in daily life for this study: the *Brief Rating List of Playfulness in Different Situations* (BRLPS). Additionally, participants provided ratings on the perceiv*ed societal appropriateness* of being playful in the given situations. This will allow for a comparison of the two perspectives.

It has already been mentioned that there is a paucity of research on playfulness in Eastern countries (cf. Yue et al., 2016). However, a few studies exist that should be highlighted. The first translation of the word “playfulness” in Chinese emerged in Taiwan. Researchers used the word 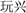 (wanxing),” which means “being in the mood to play” or “having an interest in playing”. Yu and her colleagues (Yu et al., 2003) discuss the influence of traditional Chinese values on people’s attitudes toward playfulness, such as “play only belongs to children” and “adults should work hard and be serious.” However, they also noted that because of globalization and the impact of a post-materialist value system, playfulness is becoming more and more important among young Taiwanese (Yu et al., 2003). Those who have fun at work experience high spontaneity, concentration, relaxation and happiness, which contributes to creativity, team feelings and better work performance (Yu, 2004). Their definition of playfulness is: “… a personal characteristic of pleasantry temperament, combining physical, cognitive and social spontaneity, which shows the power to begin energetically or to concentrate on events or activities, and the ability to utilize resources in solving problems or in rising to the challenge of own competence” (Yu et al., 2007; p. 416). Based on this definition, Yu and her colleagues developed an *Adult Playfulness Questionnaire* (APQ, Yu et al., 2003) within the context of Eastern culture. In total, 755 Taiwanese adults from different occupations were consulted, and the items were derived from a literature review, group discussion, open questionnaires, and in-depth interviews. The results showed acceptable reliability and validity, and factor analysis yielded a six-factor model (Yu et al., 2003). Later, the authors favored a reduced three-factor model of adult playfulness; namely, “pleasantry,” “initiative and concentration,” and “creativity” (Yu et al., 2007). It is important to note that the term “pleasantry” is being used in a different sense here compared to the common understanding. Yu et al. (2007) argue that it is a combination of a sense of humor and a childlike manner. We kept the original translation by Yu and colleagues because we wanted to keep the terminology of the original authors.

In a review article, Li (2006) noted that playfulness contributes positively to the creativity of college students. Zhang (2011, Unpublished) developed a measure of playfulness for college students that consists of a seven-factor structure: namely, sense of humor, creativity, curiosity, activity, sociality, spontaneity, and pleasure. Differences in playfulness were found for gender (males scored higher than females in creativity, whereas females were higher in spontaneity, sociality, and pleasure); grades (e.g., first-years showed the highest level of playfulness); majors (e.g., literature and history students were higher than science and engineering students in sense of humor); and backgrounds (e.g., students from the city scored higher than students from rural areas). A recent study used two student samples from Hong Kong and Guangzhou (China) and showed the relationship between playfulness and their humor styles. The results suggested that highly playful Chinese students preferred using affiliative and self-enhancing humor to amuse themselves and others (Yue et al., 2016).

One recent study (Barnett, 2017) addressed the cultural aspect of playfulness by comparing three groups of Chinese female graduate students who varied in the length of time they had lived in the United States, and thus had been exposed to American culture, with a fourth group of American students who were born in the United States and had always lived there. Her findings suggest that playfulness can be culturally transmitted to Chinese women who are from a different culture. However, to the best of our knowledge, there are no direct comparisons of adult playfulness in Western and Eastern cultures.

#### The Present Study

The aim of the current study was threefold. First, we aimed to establish measurement equivalence of two playfulness instruments, one of which was developed in Switzerland (i.e., *Short Measure of Adult Playfulness*, SMAP; Proyer, 2012b), and one in Taiwan (*Adult Playfulness Questionnaire*, APQ; Yu et al., 2003). Second, we aimed to investigate cross-cultural playfulness by comparing mean level differences of playfulness between students from the West (German-speaking countries) and the East (mainland China). Chinese students were expected to be less playful in comparison to German-speaking students using both measures, Western and Eastern. Third, we aimed to explore the cross-cultural differences of playfulness in different situations and to estimate the social appropriateness of playfulness in these situations.

## Materials and Methods

### Participants

Sample 1 consisted of 143 German-speaking students aged 18–48 years (*M* = 23.2, *SD* = 4.6) from Switzerland (*n* = 100), Germany (*n* = 31) and Austria (*n* = 12). Of these, 72.0% were female (*n* = 103). Approximately two-thirds were single (66.4%) and slightly less than a third were in a relationship or married (32.9%). About a third held a Bachelor of Science degree from a university (31.5%); of the rest, 67.1% held a school-leaving diploma qualifying for attending university, and 1.4% had completed compulsory education.

Sample 2 consisted of 176 university students who were aged 18–24 years (*M* = 19.8, *SD* = 1.2) and lived in Guangzhou, mainland China. Of these, 56.3% were female (*n* = 99). Three-quarters of the participants were single (*n* = 132, 75.0%) while 22.2% (*n* = 39) were in a relationship; the 5 remaining participants did not indicate their marital status. Almost all participants held a university degree (Bachelor of Science) or were currently enrolled at a university (*n* = 169, 96.0%).

Sample 3 consisted of 100 university students aged 18–27 years (*M* = 20.4, *SD* = 1.5) and living in Beijing, mainland China. Of these, 69% were female (*n* = 69). The majority of the participants (*n* = 83, 83.0%) were single. Almost all of them held a university degree (Bachelor of Science) or were currently enrolled at a university (*n* = 95, 95.0%).

### Instruments

#### Short Measure of Adult Playfulness (SMAP)

The SMAP (Proyer, 2012b) consists of five items that allow for a global assessment of adult playfulness. Answers are given on a 7-point Likert scale ranging from 1 = “*strongly disagree*” to 7 = “*strongly agree*”. All items are positively keyed. Previous data (e.g., Proyer, 2012b; Proyer and Ruch, 2011) showed a one-dimensional solution with satisfactory reliabilities (Cronbach’s α > 0.80). The SMAP also converges well with other measures of playfulness (Glynn and Webster, 1992, 1993; Barnett, 2007) and the need for play (Jackson, 1974). High scorers in the SMAP expressed higher approval and liking of an unstructured working environment and higher approval and liking of an abstract painting in comparison with low scorers who expressed greater disapproval of the unstructured work space and an abstract art piece; no differences were found in rating for an orderly work space and simple geometric figures (Proyer, 2012b). The Chinese version of the SMAP (SMAP-CN) was developed for the current study using the back-translation procedure (see below). It consists of the same items and scoring rules as the German version. A sample item is 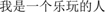 (I am a playful person)”. We used the term 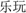
” as a translation for playful because it can reduce the negative linguistic bias of the current translation (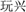
”) by the Taiwanese scholars. The SMAP-CN can be found in the online Supplementary Materials of the study.

#### Adult Playfulness Questionnaire (APQ)

The APQ scale (Yu et al., 2003) consists of 29 items loading on three factors: “*pleasantry*,” “*initiative and concentration*,” and “*creativity*”. All items are positively keyed and utilize a five-point Likert scale (1 = “*strongly disagree*,” 5 = “*strongly agree*”). Yu and her colleagues (Yu et al., 2003) reported a satisfying internal consistency (Cronbach α = 0.95 for the total score) and acceptable construct, concurrent, and discriminant validities. One sample item of the Chinese version (Yu et al., 2003) is 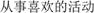, 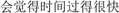, 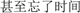 (For whatever I love to do, time will fly by and I even forget about the time spent on it)”. A German translation of the items has been used in a previous study (Proyer and Jehle, 2013).

#### Brief Rating List of Playfulness in Different Situations (BRLPS)

The BRLPS was developed for this study to assess playfulness in different situations. It consists of 14 different contexts with two perspectives: the self-perspective and the perceived society perspective. The self-perspective covers the level of playful behavior expressed by participants when they are with certain people (e.g., friends, family), or when they are in certain situations (e.g., at the workplace). The perceived society perspective covers the perception of how society would rate the appropriateness of being playful around these people or in the given situation. Answers are given on a 7-point scale ranging from 1 = “*Not at all*” to 7 = “*Very much*” and also include “*Not applicable.*” The Chinese version of the scale was developed for the current study and had the identical contexts as well as scoring rules with the German version. Two sample situations are: in German, “zusammen mit Grosseltern (together with grandparents)” and “in der Öffentlichkeit (in public)”; in Chinese, 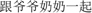 (together with grandparents)” and 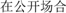 (in public)” The German and Chinese versions used in this study are provided in the online Supplementary Materials.

### Procedure^1^

#### Translation

The Short Measure of Adult Playfulness (SMAP; Proyer, 2012b) was translated from English into Chinese using Brislin’s (1970) back-translation model. The first author of the current study did the initial translation from Chinese into German. Afterward a master student who studied psychology in mainland China back-translated all the items independently. The two versions of the instruments were compared for concept equivalence by another Chinese student who was studying for a Ph.D. in psychology at the University of Cambridge. Once an error or disagreement was found in the back-translated version, the first author tried to retranslate the item and discussed this with the original author of the scale (the second author of the current study). This procedure continued until all three translators agreed that the two versions of the instruments were identical and had no errors in meaning. As mentioned before, there was no corresponding term for playfulness in Chinese, and the Taiwanese translation 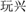 (wanxing)” could not be used because the term is not used in daily language in mainland China. Hence participants would have more than one way of understanding its meaning (e.g., it could mean interested in playing, or a trait of playing), which would lead to confusion and stronger linguistic bias. Therefore, after discussing the issue with experts as well as laypeople, playfulness was translated as 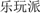 (lewanpai)” in the current study, which means a person who enjoys playing. An explanation of playfulness was presented in the introduction to the SMAP for both German-speaking participants and Chinese. Thus we ensured that all participants had an identical understanding of the concept. The Adult Playfulness Questionnaire (APQ; Yu et al., 2003) was adapted into simplified Chinese accordingly and the word 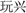 (wanxing)” was replaced with 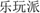 (lewanpai)” for the participants from mainland China.

#### Recruitment

We trained two undergraduate students who were studying psychology at Sun Yat-sen University (Guangzhou) and Renmin University (Beijing) to recruit the participants in mainland China in paper-pencil form. Meanwhile, a German version and a Chinese version of the questionnaires were created online through a web-based survey solution (SurveyMonkey). Advertisements were placed on the Internet and via email (e.g., students’ forums, social media, university mailing list, etc.), and in a public place such as a pin board to get as many participants as possible. As a result, we had access to students who studied in German-speaking countries (mainly Switzerland) or in mainland China. To motivate the participants, participants living in Guangzhou received a postcard as a gift, while participants who studied psychology at the University of Zurich were given 0.75 experiment-hours or a piece of sushi as an incentive for participation. Participants were not paid for their service, but were given a written feedback of individual results when interest was expressed.

#### Data Collection

All questionnaires (paper–pencil form) collected in mainland China were delivered to Switzerland by DHL and Federal Express Corporation Inc., and were then scanned using the software Remark Office OMR (version 6).

## Results

### Examination of Measurement Invariance

Although the questionnaires were translated using a translation-back-translation procedure, measurement equivalence must be established for enabling comparisons (see e.g., Mullen, 1995; van de Vijver and Tanzer, 2004). Metric measurement invariance was tested for the SMAP and APQ (testing each facet separately) using a multi-group CFA with the lavaan (Rosseel, 2012) and semTools packages (semTools Contributors, 2015) in *R*. It was tested by forcing all item^2^ loadings to be equal across groups. This model was then compared with the baseline model that allows a free estimation of the item loadings, comparing the difference in the CFI and the RMSEA. Changes of ≤|0.01| in the CFI and changes of ≤|0.015| in the RMSEA were used as cut-offs to indicate measurement invariance, based on the recommendations by Cheung and Rensvold (1999) and Chen (2007). Metric measurement invariance was tested across the three samples. The results are displayed in **Table 1**, which depicts the fit indices of the baseline model (in which the item loadings were allowed to vary freely), the metric invariance model (in which the item loadings were constrained to be equal across groups), and the changes in the CFI and the RMSEA.

**Table 1:** Fit indices of models assessing metric (fixed loadings) invariance of SMAP and APQ across three samples.

Measurement invariance models	*df*	χ^2^	CFI	RMSEA	CFI change	RMSEA change
**SMAP**						
Baseline model	15	39.63	0.97	0.11	–	–
Metric invariance	23	47.45	0.98	0.09	0.000	0.021
**APQ**						
Pleasantry						
Baseline model	60	318.43	0.82	0.18	–	–
Metric invariance	74	337.70	0.82	0.17	0.004	0.017
Initiative and Concentration						
Baseline model	27	163.88	0.90	0.20	–	–
Metric invariance	37	189.52	0.88	0.18	0.012	0.020
Creativity						
Baseline model	6	20.96	0.97	0.14	–	–
Metric invariance	12	38.17	0.95	0.13	0.022	0.009


*χ^*2*^, chi square. CFI, comparative fit index, RMSEA, root mean square error of approximation. SMAP, Short Measure of Adult Playfulness. APQ, Adult Playfulness Questionnaire.*

As shown in **Table 1**, the baseline model had an adequate fit to the data for SMAP and the creativity facet of the APQ. However, the remaining facets of the APQ had a rather weak fit to the data. The CFI changes were <|0.01| for the SMAP and pleasantry and the RMSEA changes were <|0.015| for creativity. Follow-up analyses were conducted for assessing partial measurement invariance of the APQ, comparing the metric invariance of each of the items in the three samples. The metric invariance was supported for each item in all three facets of the APQ, as the CFI change between the baseline model and the metric invariance model was <|0.01| (with a range from |0.000| to |0.008|). Thus, partial measurement invariance was supported in our study and this allows us to meaningfully compare the mean level differences between the playfulness scores across the samples^3^.

### Correlations Among the Playfulness Measures and Situational Ratings of Playfulness

In the next step, to test for overlaps and to establish validity of these measures, we correlated the scores obtained on the two measures of playfulness in each sample. The results are presented in **Table 2**. The correlation coefficient between the total score of the APQ and the SMAP was 0.61 for the German-speaking sample, and 0.46 for both Chinese samples. Of the subscales of the APQ, pleasantry correlated highest with the SMAP (coefficients ranged from 0.43 to 0.69), while the other two facets correlated numerically much lower with the SMAP. The correlation coefficients between ‘initiative and concentration’ and the SMAP varied across the three samples (from 0.11 to 0.37), while creativity demonstrated robust associations with the Guangzhou sample (0.30) and the German sample (0.37), but the coefficient was lower for the Beijing sample (0.11).

**Table 2:** Correlations between SMAP and APQ in three samples (Controlled for Gender).

	SMAP		
	
	Sample 1 (*n* = 116)	Sample 2 (*n* = 164)	Sample 3 (*n* = 91)
**APQ**			
Total	0.61^∗∗∗^	0.46^∗∗∗^	0.46^∗∗∗^
Pleasantry	0.69^∗∗∗^	0.43^∗∗∗^	0.52^∗∗∗^
I&C	0.30^∗∗^	0.37^∗∗∗^	0.11
Creativity	0.21^∗^	0.27^∗∗∗^	0.36^∗∗∗^


*SMAP, Short Measure of Adult Playfulness. APQ, Adult Playfulness Questionnaire. I&C, Initiative & Concentrating. Sample 1, German-speaking sample; Sample 2, Guangzhou sample, and Sample 3, Beijing sample. ^∗^*p* < 0.05, ^∗∗^*p* < 0.01, ^∗∗∗^*p* < 0.001, two-tailed.*

When correlating the situational ratings of playfulness with the two measures of playfulness, the coefficients for SMAP and APQ were largely around 0.30 (see **Table 3**). The self-ratings of different situations for the German-speaking sample showed a median of *r* = 0.33 for SMAP and 0.24 for APQ; the Beijing sample showed a median of *r* = 0.31 for both; and the Guangzhou sample was numerically smaller (median *r* = 0.12 for SMAP and 0.23 for APQ). When analyzing the perceived society perspective, they were uncorrelated in the German-speaking sample while there were some associations in the two Chinese samples (see **Table 4**; e.g., the “with parents” situation). These results reveal that in a collectivistic country like China, the perceived society norms had an impact on the associations with playfulness.

**Table 3:** Correlations between SMAP, APQ and situational ratings of playfulness (self-perspective).

	Sample 1		Sample 2		Sample 3	
			
	SMAP	APQ	SMAP	APQ	SMAP	APQ
With grandparents	0.04	0.02	0.28**	0.15	0.22*	0.08
With parents	0.24**	0.20*	0.31**	0.23**	0.43***	0.31**
With siblings	0.27**	0.33**	0.28**	0.24**	0.41***	0.32**
With partner	0.35***	0.33**	0.29**	0.26**	0.21	0.15
With children	0.10	0.12	0.12	0.29**	0.29*	0.06
With friends	0.44***	0.40**	0.39***	0.41***	0.32**	0.27**
With classmates	0.33***	0.30**	0.38***	0.31***	0.40***	0.30**
With work colleagues	0.32**	0.33**	0.12	0.22*	0.33**	0.35**
With teachers	0.32***	0.25**	0.12	0.16*	0.35***	0.30**
With boss	0.35***	0.22*	-0.03	0.02	0.29*	0.34**
In public	0.42***	0.20*	0.11	0.29***	0.33**	0.32**
In business meeting	0.35***	0.18	-0.05	-0.01	0.27	0.56***
On online-forum	0.41***	0.20	0.06	0.19*	0.05	0.31**
On social media	0.20*	0.26**	0.08	0.23**	0.22*	0.36***


*SMAP, Short Measure of Adult Playfulness. APQ, Adult Playfulness Questionnaire. Sample 1, German-speaking sample; Sample 2, Guangzhou sample, and Sample 3, Beijing sample. ^∗^*p* < 0.05, ^∗∗^*p* < 0.01, ^∗∗∗^*p* < 0.001, two-tailed.*

**Table 4:** Correlations between SMAP, APQ and situational ratings of playfulness (society-perspective).

	Sample 1		Sample 2		Sample 3	
			
	SMAP	APQ	SMAP	APQ	SMAP	APQ
With grandparents	-0.12	-0.06	0.21**	0.12	0.28**	0.10
With parents	0.02	-0.07	0.21**	0.23**	0.34**	0.11
With siblings	0.03	-0.01	0.28***	0.26**	0.19	0.15
With partner	0.10	0.08	0.08	0.25*	0.18	0.06
With children	-0.04	0.03	0.08	0.24*	0.09	0.03
With friends	0.08	0.11	0.20**	0.31***	0.20	0.15
With classmates	-0.09	-0.17	0.17*	0.25**	0.34**	0.17
With work colleagues	0.06	0.00	-0.05	0.04	0.25*	0.37**
With teachers	0.10	0.08	0.06	0.21**	0.37**	0.30**
With boss	0.10	0.07	0.04	0.15	0.33**	0.31*
In public	0.10	-0.06	0.03	0.21**	0.22*	0.18
In business meeting	0.08	-0.03	0.22	0.21	0.33*	0.44**
On online-forum	0.04	-0.01	0.04	0.18*	-0.09	0.07
On social media	-0.02	0.05	0.12	0.24**	-0.01	0.11


*SMAP, Short Measure of Adult Playfulness. APQ, Adult Playfulness Questionnaire. Sample 1, German-speaking sample; Sample 2, Guangzhou sample, and Sample 3, Beijing sample. ^∗^*p* < 0.05, ^∗∗^*p* < 0.01, ^∗∗∗^*p* < 0.001, two-tailed.*

### Descriptive Statistics of the Scales

**Table 5** shows the descriptive statistics of the SMAP and APQ. An examination of each scale’s skewness and kurtosis suggested that they were all normally distributed in three samples. Their internal consistency was high in all three samples (all ≥ 0.71). The mean scores were comparable (where previous data was available) to prior research (Yu et al., 2003; Proyer, 2012b; Proyer and Jehle, 2013; Yue et al., 2016). We also checked whether they correlated with gender, age, and collection mode (paper and pencil vs. online). Correlation coefficients with age and collection mode were negligible, but there were minor associations with gender (all < 5% overlapping variance). Nevertheless, we decided to control for the potential effects of gender in the analyses conducted subsequently.

**Table 5:** Psychometric characteristics and correlations with gender of SMPA and APQ in three samples.

	Sample 1						Sample 2						Sample 3					
			
	*M*	*SD*	α	*S*	*K*	*r*_sex_	*M*	*SD*	α	*S*	*K*	*r*_sex_	*M*	*SD*	α	*S*	*K*	*r*_sex_
**SMAP**																		
Total	5.03	1.10	0.85	-0.99	0.85	-0.17^∗^	4.86	1.13	0.82	-0.83	1.00	-0.09	5.02	1.13	0.82	-0.48	-0.61	-0.04
**APQ**																		
Total	3.87	0.47	0.84	-0.73	0.46	-0.17	3.69	0.65	0.91	-0.30	-0.08	0.01	3.83	0.63	0.90	-0.60	0.85	-0.13
Pleasantry	3.95	0.64	0.83	-0.88	0.65	-0.04	3.63	0.81	0.88	-0.29	-0.40	-0.03	3.75	0.80	0.88	-0.52	-0.34	-0.10
I&C	4.09	0.57	0.79	-0.48	-0.32	-0.20^∗^	4.10	0.73	0.91	-0.85	0.58	0.12	4.23	0.72	0.87	-0.85	0.25	-0.01
Creativity	3.36	0.71	0.71	-0.19	0.32	-0.20^∗^	3.18	0.87	0.83	-0.28	-0.34	-0.04	3.39	0.94	0.86	-0.06	-0.66	-0.22^∗^


*SMAP, Short Measure of Adult Playfulness. APQ, Adult Playfulness Questionnaire. I&C, Initiative & Concentrating. Sample 1, German-speaking sample (116 ≤*n* ≤ 125); Sample 2, Guangzhou sample (165 ≤*n* ≤ 168), and Sample 3, Beijing sample (92 ≤*n* ≤ 94). *M*, mean; *SD*, standard deviation; α, Cronbach alpha; *S*, Skewness; *K*, Kurtosis; *r*_*sex*_, Correlation with gender (1 = “male,” 2 = “female”). ^∗^*p* < 0.05, ^∗∗^*p* < 0.01, ^∗∗∗^*p* < 0.001, one-tailed.*

### Cross-Cultural Differences in Playfulness

In order to explore differences in playfulness between German-speaking participants and Chinese participants, a one-way analysis of covariance (ANCOVA) was conducted (covariate: gender). The independent variable “*Region*” involved three levels: German-speaking participants, participants from Guangzhou, and participants from Beijing. The dependent variables were the playfulness scores in the SMAP and APQ. The preconditions for the ANCOVA were met. In particular, the homogeneity of the regression effect was evident for the covariate, and the covariate was linearly related to the dependent measure. The results are displayed in **Table 6**.

**Table 6:** Mean level differences of playfulness in three samples.

	Sample 1			Sample 2			Sample 3			Variance	
				
	*n*	*M*	*SD*	*n*	*M*	*SD*	*n*	*M*	*SD*	*F*	*p*
**SMAP**	143	5.03	1.10	175	4.85	1.13	98	5.01	1.13	1.59	0.205
**APQ**	120	3.86_a_	0.47	168	3.68_ab_	0.65	94	3.83_b_	0.63	4.22	0.015
Pleasantry	119	3.95_a_	0.64	168	3.63_a_	0.81	96	3.75_b_	0.80	6.52	0.002
I&C	120	4.09	0.57	168	4.11	0.73	96	4.25	0.72	1.80	0.166
Creativity	120	3.36_a_	0.71	168	3.18_ab_	0.87	96	3.39_b_	0.93	3.37	0.035


*SMAP, Short Measure of Adult Playfulness. APQ, Adult Playfulness Questionnaire. I&C, Initiative & Concentrating. Sample 1, German-speaking sample; Sample 2, Guangzhou sample; Sample 3, Beijing sample. Means in a row sharing subscript are statistically different from each other at *p* < 0.05 (two-tailed) utilizing planed contrast (when comparing German-speaking sample with the two Chinese sample separately) and the Fisher’s least significant difference (LSD) procedure (when comparing the two Chinese samples). For all measures, higher means indicate higher playfulness scores.*

The table shows that the main effect of the variable *Region* for the SMAP (Proyer, 2012b) was not significant (*F*[2,412] = 1.59, *p* = 0.205). The main effect of *Region* for the total score of the APQ (Yu et al., 2003) was significant (*F*[2,378] = 4.22, *p* = 0.008, $\eta_{p}^{2}$ = 0.02), as well as being significant for the subscales *Creativity* and *Pleasantry*. Comparisons revealed that the German-speaking participants scored higher in the total score of APQ than the Guangzhou participants (Cohen’s *d* = 0.32), but did not differ from the Beijing participants. Similar results were found for the subscales *Creativity* and *Pleasantry* (Cohen’s *d* = 0.23 and 0.44, respectively) when comparing the German-speaking sample and the Guangzhou sample. A *post hoc* test (Fisher’s LSD) was conducted to explore the potential difference within mainland China (Guangzhou sample vs. Beijing sample). We found that the Beijing sample scores were higher on the total score of APQ (Cohen’s *d* = 0.23) and on the *Creativity* subscale (Cohen’s *d* = 0.23).

### Mean Level Differences of Playfulness in Different Situations

We averaged the responses of the *Brief Rating List of Playfulness in Different Situations* (BRLPS; Pang and Proyer, 2013a,b, Unpublished). In order to group the situations into different categories, we conducted a principal component analysis (PCA) on the 15 situations with oblique rotation. The eigenvalues for the first five components were 4.53, 2.63, 1.66, 1.12, and 0.89 (self-reported playfulness in given situations) and 5.20, 2.96, 1.54, 0.98, and 0.86 (perceived societal perspective). Three factors were extracted in both analyses and tentatively labeled as (a) *private situations* (e.g., with relatives and friends); (b) *formal situations* (e.g., with work colleagues and teachers); and (c) *university/online settings* (e.g., online forum and social media). A one-way analysis of covariance (ANCOVA) was conducted (covariate: gender) and *post hoc* tests (Fisher’s LSD) were used for pairwise comparisons after obtaining significant differences. **Table 7** (self-perspective) and **Table 8** (perceived society perspective) show the sample size (*n*), mean score (*M*), standard deviation (*SD*), and findings of the ANCOVA (*F* score and *p*-value).

**Table 7:** Mean level differences of playfulness in different situations in three samples (self-perspective).

	Sample 1			Sample 2			Sample 3			Variance	
				
	*n*	*M*	*SD*	*n*	*M*	*SD*	*n*	*M*	*SD*	*F*	*p*
**Private situation**	139	5.43_a_	0.78	175	4.95_ab_	1.05	95	5.24_b_	0.94	8.91	<0.001
With grandparents	122	3.87	1.74	166	3.58	1.75	90	3.88	1.70	0.90	0.205
With parents	137	4.87_a_	1.51	175	4.35_a_	1.60	95	4.62_b_	1.68	3.20	0.021
With siblings	121	5.92_ab_	1.09	160	5.33_a_	1.38	87	5.55_b_	1.11	8.36	<0.001
With partner	104	6.21_a_	1.11	108	5.86_a_	1.29	64	6.14_b_	0.99	2.58	0.039
With children	103	5.82	1.19	91	5.48	1.37	57	5.79	1.24	1.52	0.110
With friends	135	5.99	0.97	173	5.99	1.06	95	6.09	0.90	0.44	0.321
**Formal situation**	139	3.38	1.18	174	3.66	1.06	95	3.67	1.19	2.26	0.105
With work colleagues	117	4.40	1.54	109	4.47	1.24	60	4.47	1.32	0.62	0.470
With teachers	129	2.39_ab_	1.38	174	3.37_a_	1.42	94	3.49_b_	1.47	21.28	<0.001
With boss	122	2.73	1.54	106	3.09	1.55	61	3.18	1.51	2.21	0.056
In public	139	4.40	1.48	174	4.15	1.29	94	4.09	1.33	2.06	0.065
In business meeting	99	2.35_ab_	1.35	82	2.90_a_	1.64	50	3.02_b_	1.55	4.28	0.008
**University/online settings**	139	4.63_ab_	1.22	175	5.12_a_	1.37	95	5.16_b_	1.01	8.30	<0.001
With classmates	136	5.44_ab_	1.15	174	5.22_a_	1.08	95	5.18_b_	1.19	2.68	0.035
On online-forum	85	3.64_a_	1.78	127	4.87_a_	1.59	74	5.23_a_	1.31	22.99	<0.001
On social media	119	4.08_ab_	1.61	170	5.19_a_	1.44	93	5.19_b_	1.33	22.69	<0.001


*Sample 1, German-speaking sample; Sample 2, Guangzhou sample; Sample 3, Beijing sample. The different participant number is due to the fact that the situation items did not apply for everyone in the sample and we offered them the option “it doesn’t apply to me” for each situation. For instance, because most of the participants are students and it is difficult for some of them to judge the situation with children, they would decide to answer “it doesn’t apply to me.” Therefore, different situations end up with different participant numbers. Means in a row sharing subscript are statistically different from each other at *p* < .05 (one-tailed) according to Fisher’s least significant difference (LSD) procedure. For all measures, higher means indicate higher playfulness scores.*

**Table 8:** Mean level differences of playfulness in different situations in three samples (perceived society perspective).

	Sample 1			Sample 2			Sample 3			Variance	
				
	*n*	*M*	*SD*	*n*	*M*	*SD*	*n*	*M*	*SD*	*F*	*p*
**Private situation**	132	5.63_a_	1.02	171	5.15_a_	1.10	92	5.45_b_	0.92	6.80	<0.001
With grandparents	120	4.67_a_	1.66	161	4.12_ab_	1.75	87	4.68_b_	1.58	3.61	0.014
With parents	131	5.03_a_	1.34	168	4.64_ab_	1.65	92	5.02_b_	1.43	2.51	0.042
With siblings	120	6.06_ab_	1.15	156	5.49_a_	1.47	83	5.72_b_	1.05	6.44	0.001
With partner	121	5.76	1.22	104	5.82	1.17	62	5.89	1.07	0.34	0.358
With children	115	6.31_ab_	1.17	92	5.68_a_	1.24	59	5.81_b_	1.27	6.87	0.001
With friends	128	6.03	1.08	170	5.90	1.12	91	6.01	1.01	0.47	0.312
**Formal situation**	132	2.75_ab_	1.15	171	4.03_a_	1.18	92	4.28_b_	1.24	47.94	<0.001
With work colleagues	124	3.81_ab_	1.61	105	4.67_a_	1.44	63	4.94_b_	1.28	15.57	<0.001
With teachers	125	2.06_ab_	1.47	169	3.98_a_	1.68	91	4.20_b_	1.55	67.66	<0.001
With boss	120	2.08_ab_	1.46	105	3.64_a_	1.76	61	3.89_b_	1.72	36.47	<0.001
In public	132	3.52_ab_	1.53	171	4.27_a_	1.48	90	4.49_b_	1.50	15.16	<0.001
In business meeting	111	1.81_ab_	1.25	83	3.41_a_	2.05	49	3.76_b_	1.94	32.29	<0.001
**University/online settings**	131	4.79_ab_	1.25	171	5.28_a_	1.16	92	5.17_b_	1.02	12.70	<0.001
With classmates	131	5.24	1.31	170	5.42	1.21	92	5.52	1.24	1.48	0.115
On online-forum	105	4.12_a_	1.75	124	5.01_a_	1.61	72	5.61_a_	1.30	19.96	<0.001
On social media	121	4.83_ab_	1.40	166	5.39_a_	1.40	90	5.66_b_	1.29	10.30	<0.001


*Sample 1, German-speaking sample; Sample 2, Guangzhou sample; Sample 3, Beijing sample. The different participant number is due to the fact that the situation items did not apply for everyone in the sample and we offered them the option “it doesn’t apply to me” for each situation. For instance, because most of the participants are students and it is difficult for some of them to judge the situation with children, they would decide to answer “it doesn’t apply to me.” Therefore, different situations end up with different participant numbers. Means in a row sharing subscript are statistically different from each other at *p* < 0.05 (one-tailed) according to Fisher’s least significant difference (LSD) procedure. For all measures, higher means indicate higher playfulness scores.*

#### Self-Perspective

As displayed in **Table 7**, the three samples differed in their ratings of private situations (*F*[2,405] = 8.91, *p* < 0.001) and of university/online settings (*F*[2,387] = 29.08, *p* < 0.001). However, no differences were found in the formal situations (*p* = 0.075, one tailed). *Post hoc* tests showed that, in comparison with the Guangzhou sample, participants from the German-speaking sample and the Beijing sample seemed to be more playful in their private life (Cohen’s *d* = 0.52 and 0.29 respectively). Additionally, the German-speaking sample scored lower in playfulness than both the Guangzhou sample (Cohen’s *d* = 0.79) and the Beijing sample (Cohen’s *d* = 0.89) when they were in university/online situations, whereas no differences were found between the two Chinese samples.

#### Perceived Society Perspective

As displayed in **Table 8**, from the perceived perspective of society, differences across the three samples were found in private situations (*F*[2,391] = 6.80, *p* < 0.001), formal situations (*F*[2,391] = 49.58, *p* < 0.001), and in university/online settings (*F*[2,377] = 18.05, *p* < 0.001). *Post hoc* tests showed that, in comparison with the Guangzhou sample, the German-speaking sample rated that it would be more appropriate from society’s perspective to behave playfully when they were in private situations (Cohen’s *d* = 0.45). The Beijing sample did not differ from the other two samples in their private situations from the perceived society perspective. Interestingly and unexpectedly, in comparison with both Chinese samples, participants in the German-speaking sample indicated that it would be *less* appropriate from the perspective of society to behave playfully when in formal situations (Cohen’s *d* = 1.00 for the Guangzhou sample, and Cohen’s *d* = 1.13 for the Beijing sample). No differences were found across the two Chinese samples in the formal situations from the perceived society perspective. Also, in comparison with the German-speaking sample, both Chinese samples rated that it would be more appropriate from society’s perspective to behave playfully in university/online settings (Cohen’s *d* = 0.52 for the Guangzhou sample, and Cohen’s *d* = 0.79 for the Beijing sample).

## Discussion

Recent years have seen a growing interest in the study of adult playfulness as a personality trait. To the best of our knowledge, only a few studies have taken a cross-cultural perspective into account (see Barnett, 2017), and a direct comparison of different cultures was missing. We aimed to narrow this gap in the literature by collecting data from German-speaking countries and an Eastern country (China), and by analyzing data using an instrument developed in a German-speaking country and one that has been developed in Taiwan. This allows for a more comprehensive analysis of potential differences, in contrast to using only one instrument that has been developed from a certain cultural perspective. Our expectations derived from previous literature were only partially supported as there were only a few differences across the two tested regions. Hence the differences were smaller than expected at the trait level. One might argue that future research should probably focus on the identification and analysis of inter-individual differences in playful behavior in specific situations or personal relationships, and in the perception of expectations found in societal norms, as such findings are potentially more informative about cultural differences compared to our initial study. The results, however, provide an initial overview not only of cross-cultural diversity but also of cross-cultural similarities, both of which contribute toward a better understanding of the nature of playfulness.

As expected, mean level differences in playfulness can be observed between the German-speaking sample and the Guangzhou sample with small to middle effect sizes. This could be explained by the negative bias toward play in Chinese culture. As mentioned above, play is traditionally considered as not obeying the rules and is mostly negatively connoted. However, given the effect sizes, the differences should not be over-interpreted. An observation that may be of interest for follow-up studies is that the playfulness scores of the Beijing sample were always located between the other two samples: In certain scales and certain situations (e.g., the pleasantry subscale and in university/online settings), they rated themselves similarly to the Guangzhou sample, but in other scales and other contexts they rated themselves similarly to the German-speaking sample (e.g., the creativity subscale and in private situations). This might be due to differences in the mindset between South China and North China and, therefore, within-country differences may also provide a fruitful area for future research. For example, people in North China have a flourishing tradition of enjoying “cross talk” (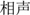; xiangsheng), which concentrates on language and word play, such as using puns, homonyms, dialects, idioms, and double entendre (Chey, 2014). This may also reflect a somewhat playful nature of people who live in the north of China and may have led to higher subjective ratings of playfulness than of people who live in the south. Hence within-country differences need consideration when thinking about playfulness in China.

Recent work has suggested that the south-north difference in mainland China mirrors the differences between collectivistic East Asia and the more individualistic Western world. Talhelm et al. (2014) proposed the so-called “*Rice Theory*” and argued that the differences seem to appear because southern China has grown rice for 1000s of years, whereas the north has grown wheat. They argue that a history of farming rice makes cultures more interdependent whereas farming wheat makes cultures more independent, and that these agricultural legacies continue to affect people in the modern world. Their findings, based on 1,162 Han Chinese participants, confirmed their assumption that rice-growing southern China is more interdependent (Talhelm et al., 2014). This is also in accordance with the current findings. One might argue that people living in South China (e.g., Guangzhou), where they have grown rice for 1000s of years, are more collectivistic and interdependent and, therefore, rated themselves lower in playfulness in this study. However, both Renmin University and Sun Yat-sen (Zhongshan) University belong to the high-status universities in China (both ranked in the top 10 in various university rankings in China) and have students from all over China. Consequently, we checked the admission numbers of the two universities at the year of data-collection for each province in China. About 53% of the admitted students at Sun Yat-sen University were from the Guangdong province, while students from the other provinces were less well represented (about 2% on average). About 11% of the admitted students at Renmin University were from Beijing, and about 3% on average from other provinces. We also checked the number of students in the wheat-rice categorization (Talhelm et al., 2014). The portion of students from the wheat culture (north of China) at Sun Yat-sen University was only about 18%, but students from the rice culture (south of China) was 63%. At Renmin University, the portion of students from the wheat culture (north of China) was 43%, while the portion of students from the rice culture (south of China) was 23%. (The percentages exclude students from the three major herding provinces and the rice-wheat border provinces.) Therefore, we could conclude that the students are still representative of the north-south difference.

Contrary to our expectations, the German-speaking participants indicated a *lower* acceptance in society to playfulness in formal situations in comparison with the Chinese participants; in particular, in work situations (e.g., business meetings) or when interacting with teachers. The differences were even larger from the perceived society perspective. This may reflect actual differences (e.g., in implicit agreements on how business meetings are conducted), but a limitation of our study must be noted at this point as we have only tested students with potentially limited experiences of business settings. Additionally, the infrastructure and atmosphere of the universities in mainland China and in German-speaking countries differ (e.g., almost all students in China live on the campus which is separated from the outside world, whereas students in German-speaking countries often live with their parents or in a shared flat in the city). This may help to explain the differences. Students in German-speaking countries may *perceive* a business setting (based on their limited experience) as more formal and structured than their Chinese counterparts. Additionally, one might argue that the rules for such meetings in German-speaking countries are potentially more implicit and take experience to understand, whereas the setting in China is more structured. Given that many students in German-speaking countries work at least part-time to help finance their education, they are presumably in low-status jobs with little room for expressions of playfulness. Additionally, it must be noted that the students from mainland China in our sample were younger and most of them were studying full time. Therefore, when the Chinese students were questioned about general situations such as “with work colleagues” or “in business meetings,” they seemed to have even less work-related experience and therefore extrapolated experience from their university lives (e.g., when thinking of colleagues from students’ associations or meetings with students’ assignment groups). In contrast, the students from German-speaking countries would potentially recall experiences from real workplaces, such as their job as barkeeper, waiter/waitress, intern, etc., which were rather low in the hierarchy of a company. Accordingly, playfulness was not encouraged in these situations because of the low level in the hierarchy. Overall therefore, these findings need to be interpreted with caution as a replication is needed involving participants with more work experience.

In our study, we used a new translation for playfulness (i.e., 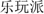 (lewanpai)”) and, therefore, had to provide a description of what this term means in the introduction to the questionnaires. This was done to ensure that all participants had an identical understanding of the concept and to avoid cultural bias. Contrariwise, however, the usage of such an explanation might reduce potential cultural differences too much in the sense that the description could have been too narrow. Although we obtained satisfactory psychometric data, more validity studies (e.g., divergent/convergent validity) of the instruments are needed in the future. This is of particular importance given that many of the measures currently in use seem to have a bias in terms of an unwanted overlap with broader personality traits—mainly emotional stability and extraversion (Proyer and Jehle, 2013; Proyer, 2017) —and lack conceptual distinctiveness from potentially related traits such as humor or creativity (e.g., Proyer et al., in press b).

Our expectation was that playfulness would be more prevalent in individualistic than in collectivistic cultures, because people in collectivistic cultures tend to display more conformity behavior than those in individualistic countries (Hofstede, 2001; p. 236). However, there are also other cultural dimensions that might play a role in explaining why German-speaking countries would score higher in playfulness than China. One candidate is the tight vs. loose culture dimension (Gelfand et al., 2011). “Tight” cultures refer to those that have strong norms and a low tolerance of deviant behavior, whereas “loose” cultures have weak norms and a high tolerance of deviant behavior. Yet both China and German-speaking countries were not on the extreme end of the scale. Future studies might aim at comparing countries with extreme dimensions (such as Pakistan vs. Netherlands). Hence, future studies might consider this variable as well as others (e.g., a comparison with English-speaking countries as they are the highest on the “loose” dimension). Our initial study shows that we can expect from such studies a contribution to the understanding of playfulness from a cross-cultural perspective.

A further limitation must be noted due to a potential confounding from an acquiescence bias. Both measures employed in this study consisted of positively worded items only. One might argue that there are differences with respect to acquiescence in the two tested countries. Smith (2004) found that acquiescence was positively related to collectivism, which supports the idea that acquiescence bias may be higher in China and may interact with (or counterbalance) the initially expected lower expressions in playfulness. It is possible therefore that our findings are biased by country-level differences in acquiescence bias, leading to an underestimation of the actual differences. However, it must be noted that data on the self-other agreement in playfulness (Ostendorf et al., 1986; Fekken et al., 1987; Proyer, 2017; Proyer and Brauer, 2018) suggest good convergence. Hence, while acquiescence may play a role and should be controlled for, self-ratings seem to reflect the perception of (well) acquainted others. There is even evidence that people can gather information on a person’s playfulness in zero-acquaintance settings (Proyer and Brauer, 2018). Nevertheless, future studies should contain reverse coded items.

Of the 29 items in the APQ (Yu et al., 2003), 11 were excluded from the current analysis mainly due to high double loadings in the factor analysis. This could be due to issues with the translation and adaptation of the items or to cultural differences. In any case, the exclusion of such a large number of items limits the interpretation of the findings. However, it must be noted that the APQ facets seem more culturally bound than might be the case in other measures (cf. Proyer and Jehle, 2013). According to an article on the construction of APQ (Yu et al., 2003), published in a Taiwanese journal, “pleasantry” is a combination of “sense of humor” and “childlike manner,” “initiative and concentration” means “flow because of intrinsic motivation,” and “creativity” stands for “solving problems with creativity” (all translations by the first author), which are all essential parts of playfulness but appear more difficult to understand in the West than in the East. The pleasantry facet is closely related to the Western understanding of playfulness (correlation coefficients ranged from 0.43 to 0.69), whereas the other two facets seem to be more embedded in Eastern thinking; nevertheless, they show some overlap. This in itself may be of interest as it points to potential differences in the understanding of the trait across more distant cultures (e.g., individualistic vs. collectivistic) and more related countries (e.g., China and Taiwan).

Participants were asked to rate their playfulness (self/perceived society perspective) generally in different situations. However, the samples were all students for the sake of comparison. Hence, their experience of workplace situations was somewhat limited, as mentioned above. Answers to this question may, therefore, refer to imagined behaviors and rules at work. There are also some specific factors that could have an impact on a person’s display of playfulness, such as the working atmosphere, the size of the company, the organizational culture, etc. Such variables have not been controlled for in this study, but may have had an impact. Additionally, there might also be shifts in the general perception of the roles that play and playfulness may have at the workplace (e.g., Petelczyc et al., 2017), and how this may permeate into different cultures. It seems more common today than in previous years to relate innovativeness and creativity to companies that foster and allow for play at the workplace; e.g., when thinking of labeling Google employees in Zurich as *Zooglers* and related newspaper headlines such as “Zooglers: Why staff are paid to play in Google’s Zurich office” (The Guardian, 2018). Along with the other suggestions for future research, it would be interesting to study such changes from a longitudinal perspective and to analyze potential differences among age groups with varying exposure to Western culture from data collected in the East. As for humor, it has been argued that this has become more appreciated by people of all ages and different backgrounds (see Yue, 2014), and a similar transition may perhaps be expected for play and playfulness. Additionally, future studies should include working professionals for a further verification of cross-cultural differences and the contribution play and playfulness may have at work (Yu et al., 2007; Petelczyc et al., 2017). Instead of using only subjective instruments, some objective measurements could be added as well, such as uploading a picture of the work desk, which can be an indicator of playfulness.

We used only a single question for being playful in online situations. However, given the rise of social media and online communication, a more fine-grained analysis of such settings seems warranted, especially as the standards of living in China are growing and the entertainment sector is starting to flourish. Phenomena such as spoofing (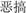; e’gao), which became more accepted in Chinese culture from early 2000 (for an overview see Yu, 2014), use parody, irony, and satire to mock those in power or make social comments. Moreover, other Internet media platforms and programs (such as PapiTube, U Can U Bibi, and Mars Intelligence Agency) enable collaboration and the production, circulation, and consumption of entertainment to be much faster, easier, and more convenient. Expressing one’s playfulness more privately on the Internet also seems to be in line with the Confucian tenets to express humor and play(fulness) in one’s daily life.

Aside from what has been mentioned earlier, this study has several limitations. Firstly, the sample sizes are comparatively small and imbalanced with respect to certain demographics. Secondly, the *Brief Rating List of Playfulness in Different Situations* was developed for this study and further studies on its validity are needed. Additionally, it represents only a selected number of persons and situations that would be worth studying in the future. Thirdly, given the size of China, it would be desirable to have even more samples to represent regional differences. Fourthly, we have tested university students only and they are probably more diligent than the general population because the two Chinese samples were from top-tier universities, which means that they scored very high during their national entrance exams, while diligence also benefits students from the University of Zurich. This hinders the generalizability of the findings and future studies should consider controlling the results for diligence. Nevertheless, one might still assume that there will be differences across, for example, certain age groups (e.g., moderated by exposure to Western culture). Finally, measurement invariance was only established for the SMAP and the creativity facet of the APQ, while there was only partial measurement invariance for the other facets. Hence findings for the APQ must be interpreted with some reservations. We have already mentioned difficulties for the cross-cultural understanding of the pleasantry facet due to translation problems; consequently, more balanced measures from a cultural perspective will be needed in follow-up studies.

## Conclusion

This study shows that it is of interest to study adult playfulness from a cross-cultural perspective (see also Barnett, 2017) and the findings have the potential to contribute toward a better understanding of the nature of playfulness. While the findings warrant replication, it seems safe to note that it would be fruitful to encourage further research on playfulness in Eastern countries (cf. Yu et al., 2003, 2007; Yue et al., 2016).

## Ethics Statement

This study was carried out in accordance with the recommendations of “the ethical guidelines of the ethics committee of the Faculty of Arts and Social Science, University of Zurich.” Participants provided either online or written informed consent in accordance with the Declaration of Helsinki.

## Author Contributions

Both authors initiated the project, designed the concepts, and analyzed the data. DP collected the data. Both authors contributed to the writing of the manuscript, read it critically and gave consent to its publication.

## Conflict of Interest Statement

The authors declare that the research was conducted in the absence of any commercial or financial relationships that could be construed as a potential conflict of interest.

## Footnotes

1 This is part of a larger data collection; other instruments have been filled out using the same sample. However, they were not relevant to the current research question and the data presented in the manuscript have not been published elsewhere.

2 All items of SMAP were used while, of the 29 items of the APQ (Yu et al., 2003), 11 were excluded from the current analysis due to high double loadings in the factor analysis.

3 The Tucker’s φ coefficient (Tucker, 1951; Lorenzo-Seva and ten Berge, 2006) was additionally computed out of Principal Component Analyses for the two measures and indicated excellent equivalence for the SMAP (all values of φ ≥ 0.997). The Tucker’s φ coefficients were excellent for the “*pleasantry*” and “*initiative and concentration*” facets of the APQ (all values of φ ≥ 0.92) facets and adequate for the “*creativity*” facet. The coefficients were φ = 0.94 between Sample 1 and Sample 2; 0.97 between Sample 2 and Sample 3; and 0.88 between Sample 1 and Sample 3.
