# Supplementary material for: An Initial Cross-Cultural Comparison of Adult Playfulness in Mainland China and German-Speaking Countries
Source: Front Psychol. 2018 Mar 29;9:421. doi: 10.3389/fpsyg.2018.00421 (PMC5885041; doi:10.3389/fpsyg.2018.00421)
Supplement: Supplementary file 1 [file Table_1.DOCX]

Supplementary Material

A cross-cultural comparison of adult playfulness in Mainland China and Switzerland

**Dandan Pang*, René Proyer**

*** Correspondence:** Dandan Pang: d.pang@psychologie.uzh.ch

# Supplementary Tables

## Table 1 Short Measure of Adult Playfulness – Chinese (SMAP-CN)

以下陈述涉及到您大体的感受、行为以及知觉，答案无所谓正确与错误。请尽可能试着用七个选项中的一个描述您习惯的行为模式和态度，并在该选项上做好标记。请使用以下选项：

（1）强烈不同意

（2）不同意

（3）稍微不同意

（4）既不同意也不反对

（5）稍微同意

（6）同意

（7）强烈同意

**请注意**：本问卷中使用的“乐玩的”这个词语译自英文"Playful"，其名词形式为乐玩派（Playfulness）。乐玩派是一种个人特质，指个体倾向于以一种想象的，非严肃、或比喻的方式去定义（或重新定义）一项活动，以增加其内在享受，投入程度以及满足感。乐玩的人即使面对无聊的情境（比如无聊的工作、学习等），也会倾向于将其重新构建成有趣好玩的。

| 重要提示:  请如此标记: 而不是: 🞊 或者 ∅ | 强烈不同意 | 不同意 | 稍微不同意 | 既不同意也不反对 | 稍微同意 | 同意 | 强烈同意 |
| --- | --- | --- | --- | --- | --- | --- | --- |
| 1. 我是一个乐玩的人。 | ⭘ | ⭘ | ⭘ | ⭘ | ⭘ | ⭘ | ⭘ |
| 1. 好朋友们会觉得我是一个乐玩的人。 | ⭘ | ⭘ | ⭘ | ⭘ | ⭘ | ⭘ | ⭘ |
| 1. 在我的日常生活中，我经常做一些乐玩的事情。 | ⭘ | ⭘ | ⭘ | ⭘ | ⭘ | ⭘ | ⭘ |
| 1. 对我来说，从严肃的心情转换到乐玩的心情并**不是**太难。 | ⭘ | ⭘ | ⭘ | ⭘ | ⭘ | ⭘ | ⭘ |
| 1. 有的时候，我沉浸在某件乐玩的事情中而完全忘记了时间。 | ⭘ | ⭘ | ⭘ | ⭘ | ⭘ | ⭘ | ⭘ |
