# Supplementary material for: An Initial Cross-Cultural Comparison of Adult Playfulness in Mainland China and German-Speaking Countries
Source: Front Psychol. 2018 Mar 29;9:421. doi: 10.3389/fpsyg.2018.00421 (PMC5885041; doi:10.3389/fpsyg.2018.00421)
Supplement: Supplementary file 2 [file Table_2.DOCX]

Supplementary Material

A cross-cultural comparison of adult playfulness in Mainland China and Switzerland

**Dandan Pang*, René Proyer**

*** Correspondence:** Dandan Pang: d.pang@psychologie.uzh.ch

# Supplementary Tables

## Table 2 Brief Rating List of Playfulness in Different Situations – Chinese (BRLPS-CN)

本页面列举了您在日常生活中可能面对的15种不同的人/情境。请您设想一下，若面对以下的人/情境，您会多大程度表现出您是乐玩的，而社会又会多大程度认为您表现出乐玩的是恰当的？请在“一点也不”到”非常”之间圈出您认为最合适的程度。若您从未遇到过该情境，请标记此项不适用于我。

| **请设想一下，若面对以下的人/情境...，** | **此项不适用于我** | **…，您会多大程度上表现出您是乐玩的？** | | | | | | | | | **…，社会在多大程度上会认为您表现出乐玩的是恰当的？** | | | | | | | | |
| --- | --- | --- | --- | --- | --- | --- | --- | --- | --- | --- | --- | --- | --- | --- | --- | --- | --- | --- | --- |
|  |  | **一点也不** |  | | | | | | | **非常** | **一点也不** |  | | | | | | | **非常** |
| 1. 跟爷爷奶奶/外公外婆在一起时 | ⭘ | ⭘ | | ⭘ | ⭘ | ⭘ | ⭘ | ⭘ | ⭘ | | ⭘ | | ⭘ | ⭘ | ⭘ | ⭘ | ⭘ | ⭘ | |
| 1. 跟父母在一起时 | ⭘ | ⭘ | | ⭘ | ⭘ | ⭘ | ⭘ | ⭘ | ⭘ | | ⭘ | | ⭘ | ⭘ | ⭘ | ⭘ | ⭘ | ⭘ | |
| 1. 跟兄弟姐妹在一起时 | ⭘ | ⭘ | | ⭘ | ⭘ | ⭘ | ⭘ | ⭘ | ⭘ | | ⭘ | | ⭘ | ⭘ | ⭘ | ⭘ | ⭘ | ⭘ | |
| 1. 跟另一半在一起时 | ⭘ | ⭘ | | ⭘ | ⭘ | ⭘ | ⭘ | ⭘ | ⭘ | | ⭘ | | ⭘ | ⭘ | ⭘ | ⭘ | ⭘ | ⭘ | |
| 1. 跟孩子在一起时 | ⭘ | ⭘ | | ⭘ | ⭘ | ⭘ | ⭘ | ⭘ | ⭘ | | ⭘ | | ⭘ | ⭘ | ⭘ | ⭘ | ⭘ | ⭘ | |
| 1. 跟好朋友在一起时 | ⭘ | ⭘ | | ⭘ | ⭘ | ⭘ | ⭘ | ⭘ | ⭘ | | ⭘ | | ⭘ | ⭘ | ⭘ | ⭘ | ⭘ | ⭘ | |
| 1. 跟同学在一起时 | ⭘ | ⭘ | | ⭘ | ⭘ | ⭘ | ⭘ | ⭘ | ⭘ | | ⭘ | | ⭘ | ⭘ | ⭘ | ⭘ | ⭘ | ⭘ | |
| 1. 跟同事在一起时 | ⭘ | ⭘ | | ⭘ | ⭘ | ⭘ | ⭘ | ⭘ | ⭘ | | ⭘ | | ⭘ | ⭘ | ⭘ | ⭘ | ⭘ | ⭘ | |

| **请设想一下，若面对以下的人/情境...，** | **此项不适用于我** | **…，您会多大程度上表现出您是乐玩的？** | | | | | | | | | **…，社会在多大程度上会认为您表现出乐玩的是恰当的？** | | | | | | | | |
| --- | --- | --- | --- | --- | --- | --- | --- | --- | --- | --- | --- | --- | --- | --- | --- | --- | --- | --- | --- |
|  |  | **一点也不** |  | | | | | | | **非常** | **一点也不** |  | | | | | | | **非常** |
| 1. 跟老师在一起时 | ⭘ | ⭘ | | ⭘ | ⭘ | ⭘ | ⭘ | ⭘ | ⭘ | | ⭘ | | ⭘ | ⭘ | ⭘ | ⭘ | ⭘ | ⭘ | |
| 1. 跟上司在一起时 | ⭘ | ⭘ | | ⭘ | ⭘ | ⭘ | ⭘ | ⭘ | ⭘ | | ⭘ | | ⭘ | ⭘ | ⭘ | ⭘ | ⭘ | ⭘ | |
| 1. 在公开场合 | ⭘ | ⭘ | | ⭘ | ⭘ | ⭘ | ⭘ | ⭘ | ⭘ | | ⭘ | | ⭘ | ⭘ | ⭘ | ⭘ | ⭘ | ⭘ | |
| 1. 开党会 | ⭘ | ⭘ | | ⭘ | ⭘ | ⭘ | ⭘ | ⭘ | ⭘ | | ⭘ | | ⭘ | ⭘ | ⭘ | ⭘ | ⭘ | ⭘ | |
| 1. 商业会谈 | ⭘ | ⭘ | | ⭘ | ⭘ | ⭘ | ⭘ | ⭘ | ⭘ | | ⭘ | | ⭘ | ⭘ | ⭘ | ⭘ | ⭘ | ⭘ | |
| 1. 网页论坛 | ⭘ | ⭘ | | ⭘ | ⭘ | ⭘ | ⭘ | ⭘ | ⭘ | | ⭘ | | ⭘ | ⭘ | ⭘ | ⭘ | ⭘ | ⭘ | |
| 1. 社交网站（如微博，人人等） | ⭘ | ⭘ | | ⭘ | ⭘ | ⭘ | ⭘ | ⭘ | ⭘ | | ⭘ | | ⭘ | ⭘ | ⭘ | ⭘ | ⭘ | ⭘ | |
