# Supplementary material for: An Initial Cross-Cultural Comparison of Adult Playfulness in Mainland China and German-Speaking Countries
Source: Front Psychol. 2018 Mar 29;9:421. doi: 10.3389/fpsyg.2018.00421 (PMC5885041; doi:10.3389/fpsyg.2018.00421)
Supplement: Supplementary file 3 [file Table_3.DOCX]

Supplementary Material

A cross-cultural comparison of adult playfulness in Mainland China and Switzerland

**Dandan Pang*, René Proyer**

*** Correspondence:** Dandan Pang: d.pang@psychologie.uzh.ch

# Supplementary Tables

## Table 3 Brief Rating List of Playfulness in Different Situations – German (BRLPS-D)

Im folgenden Teil bitten wir Sie einzuschätzen, wie verspielt Sie sich verhalten, wenn Sie mit bestimmten Menschen zusammen sind, bzw. sich in verschiedenen Situationen befinden. Schätzen Sie auch ein, ob Sie gesellschaftliche Konventionen sehen, die hier eine Rolle spielen, ob es von „der Gesellschaft“ als (un-)angemessen gesehen wird, wenn Sie sich verspielt verhalten. Wählen Sie bitte von den sieben Antwortmöglichkeiten (von „überhaupt nicht“ bis „sehr“) diejenige Antwortmöglichkeit aus, die Ihre Verspieltheit am besten beschreibt. Wenn Sie sich noch nie in einer der beschriebenen Situation befunden haben bzw. die einzuschätzenden Personen nicht passend sind (Sie z.B. kein Kind haben), markieren Sie bitte die Antwortmöglichkeit „trifft nicht auf mich zu“.

| **Stellen Sie sich vor,** **wenn Sie sich mit den beschriebenen Personen oder in einer dieser Situationen befinden...,** | **trifft nicht auf mich zu** | **..., wie verspielt verhalten Sie sich?** | | | | | | | | | **..., wie angemessen es aus Sicht der „Gesellschaft“ ist, dass Sie sich verspielt verhalten?** | | | | | | | | |
| --- | --- | --- | --- | --- | --- | --- | --- | --- | --- | --- | --- | --- | --- | --- | --- | --- | --- | --- | --- |
|  |  | **Überhaupt nicht** |  | | | | | | | **Sehr** | **Überhaupt nicht** |  | | | | | | | **Sehr** |
| 1. zusammen mit Grosseltern | **⭘** | ⭘ | | ⭘ | ⭘ | ⭘ | ⭘ | ⭘ | ⭘ | | ⭘ | | ⭘ | ⭘ | ⭘ | ⭘ | ⭘ | ⭘ | |
| 1. zusammen mit Eltern | ⭘ | ⭘ | | ⭘ | ⭘ | ⭘ | ⭘ | ⭘ | ⭘ | | ⭘ | | ⭘ | ⭘ | ⭘ | ⭘ | ⭘ | ⭘ | |
| 1. zusammen mit Geschwistern | ⭘ | ⭘ | | ⭘ | ⭘ | ⭘ | ⭘ | ⭘ | ⭘ | | ⭘ | | ⭘ | ⭘ | ⭘ | ⭘ | ⭘ | ⭘ | |
| 1. zusammen mit Partner (in) | ⭘ | ⭘ | | ⭘ | ⭘ | ⭘ | ⭘ | ⭘ | ⭘ | | ⭘ | | ⭘ | ⭘ | ⭘ | ⭘ | ⭘ | ⭘ | |
| 1. zusammen mit Kind/ern | ⭘ | ⭘ | | ⭘ | ⭘ | ⭘ | ⭘ | ⭘ | ⭘ | | ⭘ | | ⭘ | ⭘ | ⭘ | ⭘ | ⭘ | ⭘ | |
| 1. zusammen mit Freund/en | ⭘ | ⭘ | | ⭘ | ⭘ | ⭘ | ⭘ | ⭘ | ⭘ | | ⭘ | | ⭘ | ⭘ | ⭘ | ⭘ | ⭘ | ⭘ | |
| 1. zusammen mit Studienkollegen | ⭘ | ⭘ | | ⭘ | ⭘ | ⭘ | ⭘ | ⭘ | ⭘ | | ⭘ | | ⭘ | ⭘ | ⭘ | ⭘ | ⭘ | ⭘ | |

| **Stellen Sie sich vor,** **wenn Sie sich mit den beschriebenen Personen oder in einer dieser Situationen befinden...,** | **trifft nicht auf mich zu** | **..., wie verspielt verhalten Sie sich?** | | | | | | | | | **..., wie angemessen es aus Sicht der „Gesellschaft“ ist, dass Sie sich verspielt verhalten?** | | | | | | | | |
| --- | --- | --- | --- | --- | --- | --- | --- | --- | --- | --- | --- | --- | --- | --- | --- | --- | --- | --- | --- |
|  |  | **Überhaupt nicht** |  | | | | | | | **Sehr** | **Überhaupt nicht** |  | | | | | | | **Sehr** |
| 1. zusammen mit Arbeitskollegen | ⭘ | ⭘ | | ⭘ | ⭘ | ⭘ | ⭘ | ⭘ | ⭘ | | ⭘ | | ⭘ | ⭘ | ⭘ | ⭘ | ⭘ | ⭘ | |
| 1. zusammen mit Dozent (in) | ⭘ | ⭘ | | ⭘ | ⭘ | ⭘ | ⭘ | ⭘ | ⭘ | | ⭘ | | ⭘ | ⭘ | ⭘ | ⭘ | ⭘ | ⭘ | |
| 1. zusammen mit Vorgesetztem/r | ⭘ | ⭘ | | ⭘ | ⭘ | ⭘ | ⭘ | ⭘ | ⭘ | | ⭘ | | ⭘ | ⭘ | ⭘ | ⭘ | ⭘ | ⭘ | |
| 1. in der Öffentlichkeit | ⭘ | ⭘ | | ⭘ | ⭘ | ⭘ | ⭘ | ⭘ | ⭘ | | ⭘ | | ⭘ | ⭘ | ⭘ | ⭘ | ⭘ | ⭘ | |
| 1. Im Verein | ⭘ | ⭘ | | ⭘ | ⭘ | ⭘ | ⭘ | ⭘ | ⭘ | | ⭘ | | ⭘ | ⭘ | ⭘ | ⭘ | ⭘ | ⭘ | |
| 1. In geschäftlichen Sitzung | ⭘ | ⭘ | | ⭘ | ⭘ | ⭘ | ⭘ | ⭘ | ⭘ | | ⭘ | | ⭘ | ⭘ | ⭘ | ⭘ | ⭘ | ⭘ | |
| 1. Online-Forum | ⭘ | ⭘ | | ⭘ | ⭘ | ⭘ | ⭘ | ⭘ | ⭘ | | ⭘ | | ⭘ | ⭘ | ⭘ | ⭘ | ⭘ | ⭘ | |
| 1. Social Media (z.B., Facebook) | ⭘ | ⭘ | | ⭘ | ⭘ | ⭘ | ⭘ | ⭘ | ⭘ | | ⭘ | | ⭘ | ⭘ | ⭘ | ⭘ | ⭘ | ⭘ | |
